# Effects of albumin and crystalloid priming strategies on red blood cell transfusions in on-pump cardiac surgery: a network meta-analysis

**DOI:** 10.1186/s12871-024-02414-y

**Published:** 2024-01-16

**Authors:** Tianlong Wang, Jing Wang, Mingru Zhang, Han Zhang, Qiaoni Zhang, Gang Liu, Wenhao Dong, Yuefu Wang, Bingyang Ji

**Affiliations:** 1 Department of Cardiopulmonary Bypass, Fuwai Hospital, National Center for Cardiovascular Disease, State Key Laboratory of Cardiovascular Medicine, Chinese Academy of Medical Sciences & Peking Union Medical College, No. 167 Beilishi Road, Xicheng District, Beijing, 10010 China; 2 Department of Anesthesiology, Beijing Tongren Hospital, Capital Medical University, Beijing, China; 3 Surgical Intensive Care Unit, Beijing Shijitan Hospital, Capital Medical University, Beijing, China

**Keywords:** Cardiopulmonary bypass, Albumin, Crystalloid solutions, Red blood cell transfusion

## Abstract

**Background:**

In on-pump cardiac surgery, the albumin priming strategy could maintain colloid osmotic pressure better than crystalloid solutions and reduce excessive perioperative fluid balance. However, a high-quality meta-analysis is required to compare the safety of these approaches in perioperative red blood cell (RBC) transfusions. Owing to limited direct evidence, we conducted a network meta-analysis (NMA) to increase the pool of studies and provide indirect evidence.

**Methods:**

The pre-defined primary outcomes were intraoperative and the first 24 h postoperative RBC transfusion volume in units. The pre-defined secondary outcome was postoperative blood loss (the first 24 h). We reviewed all randomized controlled trials comparing albumin, crystalloid, and artificial colloid priming strategies. Studies that only displayed pre-defined outcomes could be included. A pairwise meta-analysis was performed on studies that directly compared the pre-defined outcomes between albumin and crystalloids. Additionally, a random-effects network meta-analysis (NMA) model was employed to generate indirect evidence for the pre-defined outcomes between albumin and crystalloids.

**Results:**

The literature search identified 830 studies,10 of which were included in the final analysis. Direct meta-analysis indicated that crystalloid priming significantly decreased total perioperative RBC transfusions (MD: -0.68U; 95%CI: -1.26, -0.09U; *P* = 0.02) and intraoperative RBC transfusions (MD: -0.20U; 95%CI: -0.39, -0.01U; *P* = 0.03) compared to albumin. Postoperative RBC transfusions showed a decreasing trend in the crystalloid group; however, the difference was not statistically significant. (MD: -0.16U; 95%CI: -0.45, 0.14U; *P* = 0.30). After including indirect evidence, the NMA results continued to demonstrate a higher RBC receiving with the albumin priming strategy compared to crystalloids, although the differences did not reach statistical significance. For postoperative blood loss, direct evidence showed no significant differences between albumin and crystalloid priming strategies. However, NMA evidence displayed that albumin exist higher probability of reducing postoperative blood loss than crystalloid.

**Conclusion:**

Both direct and NMA evidence indicated that the albumin priming strategy resulted in more perioperative RBC transfusions than crystalloids. Considering the additional blood management burden, the application of an albumin-priming strategy in on-pump cardiac surgery still needs more consideration.

**Supplementary Information:**

The online version contains supplementary material available at 10.1186/s12871-024-02414-y.

## Background

The optimal choice of priming fluid for cardiovascular surgery with cardiopulmonary bypass (CPB) remains uncertain. Compared to crystalloid solutions, the colloid CPB priming strategy has a more profound effect of volume expansion, decreases positive fluid balance, and requires less intraoperative fluid replacement biological [1]. As a normal endogenous colloid, albumin has more potential strength advances than other artificial colloids such as gelatines and hydroxyethyl starch (HES):1. Albumin can maintain the structural integrity of the glycocalyx and exert positive effects on several key physiological processes, such as preserving the vascular barrier, hemostasis, anti-inflammation, and restoration of microcirculation perfusion [2, 3]. 2. Albumin can coat the fluid circuit surface, thereby diminishing the contact between blood and nonbiological materials [4]. 3. Albumin could serve as an optimal component for sustaining colloid oncotic pressure to prevent tissue edema [5, 6]. However, there is a general lack of high-quality evidence for the superiority of the albumin priming strategy over crystalloids [7].

Red blood cell (RBC) transfusions are common practice for perioperative anemia in patients undergoing on-pump cardiovascular surgery and associated with adverse outcomes [8]. Applying hemostatic drugs such as tranexamic acid, finding sensitive physiological parameters to guide RBC transfusion, and designing effective blood management measures can all reduce unnecessary red blood cell transfusion, thus contributing to the prognosis of patients undergoing CPB surgery [8–10]. However, it should be noticed that the priming fluid of CPB can cause acute hemodilution, leading to an increase in the probability of perioperative blood transfusion [11]. Therefore, how to select appropriate priming solution to minimize perioperative RBC transfusion in CPB patients is the focus of clinical research. A recent large randomized controlled trial (RCT) has shown that the albumin priming strategy leads to more RBC transfusions and blood loss than crystalloids [12]. This has raised concerns regarding the albumin priming strategy. Therefore, there is a general need for an updated high-quality meta-analysis that focuses on the impact of albumin and crystalloid priming strategies on RBC transfusions.

Owing to the lack of direct RCTs comparing the priming strategy between albumin and crystalloids in RBC transfusions, we used network meta-analysis (NMA) technique to perform direct comparisons, including albumin vs. artificial colloid and artificial colloid vs. crystalloid, thus obtaining indirect evidence for the comparisons between albumin and crystalloid priming strategies. The small amounts of direct result and more indirect results obtained by NMA will further provide evidence for the difference in the effect of albumin and crystal priming strategies on RBC transfusion in cardiovascular surgery.

## Methods

### Design

The protocol was registered with the PROSPERO International Prospective Register of Systematic Reviews (PROSPERO; CRD42023435153). The explain for any amendments of the protocol was also provided on the PROSPERO register (CRD42023435153).The study adhered to the PRISMA extension statement for NMA [13] and the Meta-Analysis of Observational Studies in Epidemiology Statement [14]. The PRISMA 2020 checklist [15] of this study is included in Supplemental Table 1.

### Eligibility criteria

The PICOS criteria table was shown in Supplemental Table 2.

#### Patients

Adult patients undergoing cardiovascular surgery with CPB.

#### Intervention/Comparator

Comparing CPB priming including human albumin or crystalloid directly or indirectly.

#### Outcomes

Primary outcomes: RBC transfusion volume in units at 24 h.

Secondary outcomes: postoperative blood loss (the first 24 h).

#### Study selection

RCT comparing at least two different classes of priming strategies among albumin, artificial colloids, and crystalloids. Furthermore, studies that only displayed pre-defined outcomes could be included.

### Search strategy

A systematic search was performed using MEDLINE, EMBASE, Web of Science, and Cochrane Library. All English articles published before 1 JULY 2023 were selected. The search strategy is shown in Supplemental Fig. 1. The retrieval field is restricted to “topic” (the title, abstract, keywords and medical subject headings). Additionally, we conducted a snowball search by screening reference lists of published systematic reviews and eligible RCTs for potential consideration of further primary RCTs.

The literature search will be conducted by three researchers. Any disagreements regarding the inclusion of studies will be resolved through a process of discussion. In case of unresolved disagreements, a senior reviewer will be consulted for further input.

### Data extraction

Two reviewers independently extracted the data. The standard data extraction form included the following general information.

Study characteristics: publication year, title, authors, contact address, country, duplicate publications and sponsoring.

Methods: randomization procedure, allocation, blinding method, duration of study, design, and statistics were used.

Patient characteristics: sex, age and BMI.

Surgery characteristics: surgery types, bypass time, ischemia time, priming volume and types.

RBC transfusion strategy: the RBC transfusion thresholds during CPB and after surgery were recorded.

Postoperative variable: ventilation time, intensive care unit (ICU) days, hospital days, postoperative acute kidney injury (AKI) rate and mortality.

Primary and secondary outcomes. Considering that most studies only presented intraoperative and postoperative RBC transfusions (the first 24 h) separately, both intraoperative and the first 24 h postoperative RBC transfusions were extracted for further analysis. Total perioperative RBC transfusions were combined intraoperatively and postoperatively (first 24 h). Only the data measured in units for RBC were retained. Postoperative blood loss was defined as blood loss or chest tube drainage within the first 24 h postoperatively.

For continuous outcomes, means with standard deviations or medians with interquartile ranges were extracted. Under the assumption of a normal distribution, we transformed the interquartile range into standard deviations according to the Cochrane Handbook for Systematic Reviews of Interventions (Part 2, Chap. 7.7.3.5). If the standard deviation was zero, the lowest standard deviation of another group within the study was used in the meta-analysis.

### Risk of bias

The methodological quality of each trial was assessed using the Cochrane Risk of Bias Tool [16]. Three members of our team independently assessed the risk of bias in the trials based on the following six domains: allocation generation, allocation concealment, blinding, completeness of outcome data, possible selected outcome reporting, and any other potential sources of bias. Additionally, funnel plots were produced to assess reporting bias.

### Direct meta-analysis

The direct meta-analysis included studies that directly compared the pre-defined outcomes between albumin and crystalloids. Continuous and dichotomous outcomes were presented as mean differences (MD) with 95% confidence intervals (CI) and odds ratios (RR) with 95% CI, respectively. For continuous data, the inverse variance model was used, whereas for dichotomous data, the Mantel-Haenszel model was used. Statistical heterogeneity was tested using the χ² test and I^2^ statistic. When there was no statistical evidence of heterogeneity (I2 < 30%, *p* > 0.1), a fixed-effects model was adopted; otherwise, a random-effects model was used.

### Network meta-analysis

**Fig. 1:**
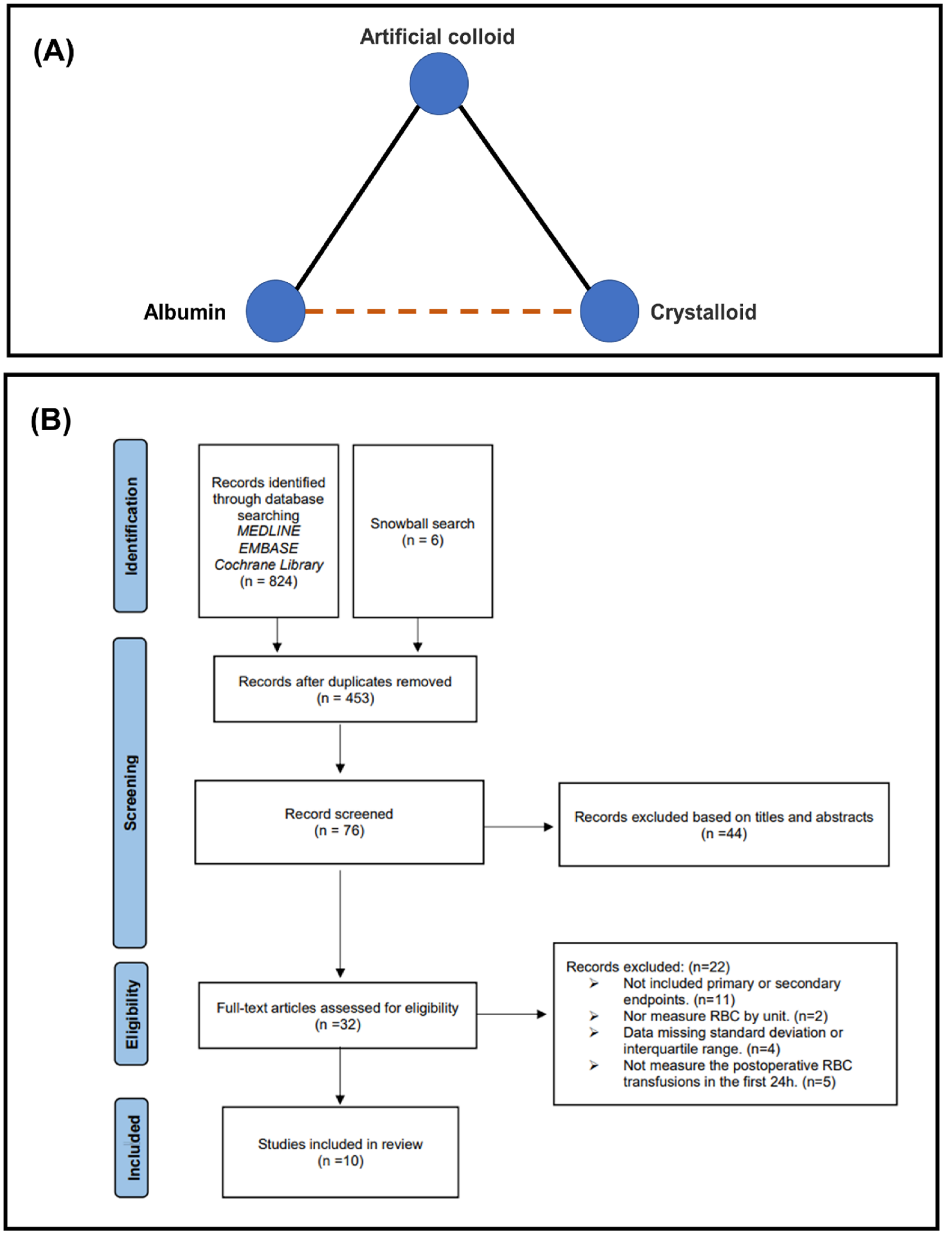
Management of the whole study. (**A**) Network map for meta-analytic priming strategies comparison: solid lines represent direct comparisons and dashed lines represent indirect comparisons. (**B**) Flow diagram for study selection

A random-effect NMA model was conducted within a Bayesian framework to combine direct and indirect evidence for the comparison between albumin and crystalloids [17]. The results were reported as MD or OR with a 95% credible interval (Crl). The surface under the cumulative ranking values (SUCRA) was calculated to hierarchically rank each priming strategy based on the probability of being the best for a given outcome, and priming strategies were ranked from best to worst based on progressively lower SUCRA [18]. The entire NMA focuses on whether there is a difference between the pre-defined outcomes of albumin and the crystal priming strategy after indirect inclusion. The network map is shown in Fig. 1A.

#### Transitivity analysis

As an extension of clinical and methodological homogeneity to comparisons across groups of studies, transitivity refers to the validity of indirect comparisons of a treatment network. To meet the transitivity assumption, we evaluated the included studies by comparing the characteristics of the population, intervention, and study design.

#### Heterogeneity analysis

Estimate the deviation of the heterogeneity variance parameter I2 and conduct a visual display to evaluate the overall heterogeneity of the NMA model [19].

#### Consistency analysis

We checked the evidence of consistency between direct and indirect analyses using node splitting analysis [20]. If *P* < 0.05, inconsistency was considered to exist between direct and indirect analyses.

#### Sensitivity analysis

Sensitivity analysis was conducted using the leave-one-out approach.

#### Meta-regression

Meta-regression was conducted to analyze the influence of the study-level variables with respect to estimates and variation in the final results.

### Statistics

Data processing and direct meta-analysis were conducted using Review Manager (version 5.3). NMA and meta-regression were performed using the package “gemtc” in R (version 4.2.2). The “anohe” and “nodesplit” functions of “gemtc” package were used for heterogeneity and consistency analyses respectively. Funnel plots were generated using the package ‘netmeta’ in R (version 4.2.2).

### Certainty assessment

The quality of each direct, indirect, and NMA estimate was rated based on the four-step approach suggested by the Grading of Recommendations Assessment, Development and Evaluation (GRADE) Working Group [21]. We rated the certainty of the directed and indirect evidence as high, moderate, low, or very low, based on study limitations, publication bias, inconsistency, indirectness, and imprecision. The final quality of the NMA effect estimates was based on a combination of direct and circumstantial evidence quality ratings.

## Results

### Search results

A PRISMA flow diagram of the systematic search process is shown in Fig. 1B. Database and snowball searches yielded 830 records. After removing duplicates, 453 records were screened. After screening, 76 abstracts were read and 32 articles were selected for full-text reading. Eleven studies were excluded because they did not access pre-specified outcomes in the study. Additionally, 11 studies were excluded because they did not meet the pre-defined data extraction criteria. Articles excluded after full-text reading are displayed in Supplemental Table 3.

Finally, 10 studies were included in the analysis. For the comparison between albumin and artificial colloid, 4 studies were found [12, 22–24]; for the comparison between artificial colloid and crystalloid, 5 studies were included [23–27]; for the comparison between albumin and crystalloid, 5 studies were found [23, 24, 28–30]. A summary of the included studies is shown in Table 1.

**Table 1:** Details of included studies

Study	Number of patients	Cardiac surgery type	Total prime volume (mL)	Albumin group	Artificial Colloid group	Crystalloid group	Additional priming solutions	Intraoperative RBC transfusion (units)	Postoperative RBC transfusion (the 1st 24 h; units)	Total perioperative red blood cells transfusions (until 1st 24 h after surgery; units)	Postoperative blood loss or chest tube drainage	RBC transfusion thresholds
Ohqvist 1981 [1]	14	Valve	2000	20% albumin 200 ml + Ringers 1800 ml	-	Ringers 2000 ml	-	-	-	-	✔	-
Marelli 1989 [2]	100	CABG; Valve	-	Additional 200 ml 25% albumin	-	Ringers	-	✔	-	-	-	-
Kuitunen 1993 [3]	30	CABG	2000	-	HES 6% 20 ml/kg + Ringers	Ringers 2000 ml	-	✔	-	-	-	During CPB: Hct > 20%; Postoperative criteria: Hct > 30%.
Scott 1995 [4]	93	CABG	2000 (1600 for patients < 60 kg)	4.6% albumin 1000 ml + Plasmalyte 1000 ml	Polygeline 1000 ml + Plasmalyte 1000 ml	Plasmalyte 2000 ml	-	-	-	-	Blood loss	During CPB: Hct > 18%; Postoperative criteria: not applied.
Tamayo 2008 [5]	44	CABG	1750	-	Gelatin 1000 ml + Ringers 500 ml	Ringers 1500 ml	20% mannitol 100 ml; aprotinin 100 ml; 8.4% sodium bicarbonate 50 ml; heparin 5000IU	-	-	-	Blood loss	During CPB: Hct was maintained at 20–25%; Postoperative criteria: not applied.
Cho 2014 [6]	36	CABG; Valve; Aortic	-	5% albumin 500 ml + Plasmalyte 1000 ml	6% HES 130/0.4 500 ml + Plasmalyte 1000 ml (max 20 ml/kg.day HES)	-	20% mannitol 5mL/kg; sodium bicarbonate 40mEq; heparin 10 mg/L	✔	✔	-	Blood loss	During CPB: Hct > 20%; Postoperative criteria: Hct > 25%.
Skhirtladze 2014 [7]	236	CABG; Valve; Aortic	1500	5% albumin (max 50 ml/kg.day) + Ringers	6% HES 130/0.4 (max 50 ml/kg.day) + Ringers	Ringers 1500 ml	20% mannitol 100 ml; heparin 5000 IU	✔	✔	✔	Chest tube drainage	During CPB: Hb > 7 g/dL; Postoperative criteria: Hb > 8-9 g/dL.
Yanartas 2015 [8]	132	CABG	1500	-	6% HES 130/0.4 10 ml/kg + Ringers 10 ml/kg	Ringers 20 ml/kg	20% mannitol 0.5 g/kg; 7.5% sodium bicarbonate 1 ml/kg; heparin 150IU/kg	✔	✔	-	Chest tube drainage	During CPB: Hct was maintained at 22–28%; Postoperative criteria: Hct was maintained at 30%.
Maleki 2016 [9]	60	CABG	1500	5% albumin 500 ml + 0.9% NaCl 1000 ml	6% HES 130/0.4 500 ml + 0.9% NaCl 1000 ml	-	20% Mannitol 5mL/kg; sodium bicarbonate 45mEq; heparin 10 mg/L	-	-	✔	Blood loss	During CPB: Hb > 7 g/dL; Postoperative criteria: Hct > 21%.
Talvasto 2023 [10]	1386	CABG; Valve; Aortic	1500	20% albumin 300 ml + 1200 ml Ringers	-	Ringers 1500 ml	-	✔	✔	✔	-	Based on clinical judgement

### Direct meta-analysis

#### RBC transfusions

**Fig. 2:**
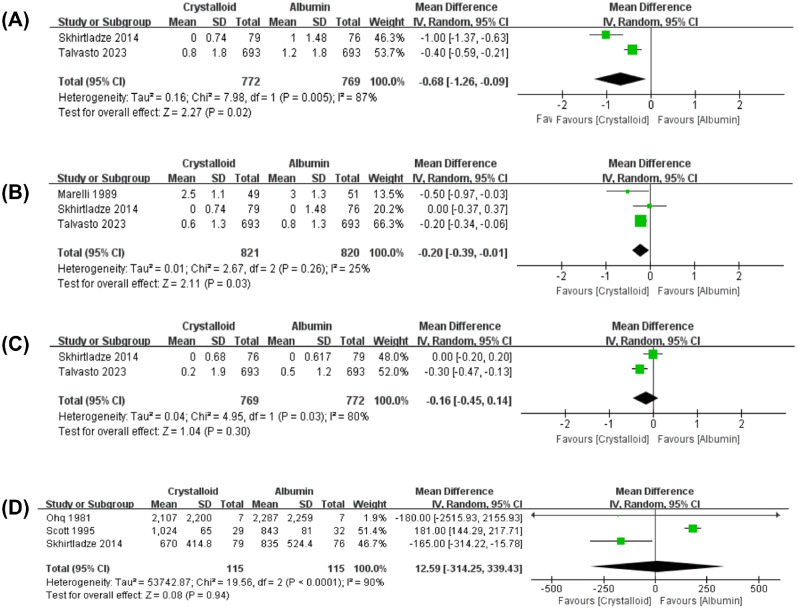
Direct meta-analysis. (**A**) Total perioperative red blood cells transfusions (until the first 24 h after surgery). (**B**) Intraoperative red blood cells transfusions. (**C**) Postoperative red blood cells transfusions during the first 24 h. (**D**) Postoperative blood loss or chest tube drainage during the first 24 h. CI, Confidence Interval

Two studies compared the total perioperative RBC transfusions (up to the first 24 h after surgery) between albumin and crystalloids [12, 24]. Detailed intraoperative RBC transfusions and postoperative RBC transfusions were also reported in these two articles. In addition, Marelli et al. presented the intraoperative RBC receiving comparing albumin and crystalloid solutions [22]. The results are shown in Fig. 2. Overall, total perioperative RBC transfusions decreased in crystalloids compared with albumin (Fig. 2A, MD: -0.68U; 95%CI: -1.26, -0.09U; *P* = 0.02). Furthermore, intraoperative RBC transfusions were significantly reduced in the crystalloid group (Fig. 2B, MD: -0.20U; 95%CI: -0.39, -0.01U; *P* = 0.03). Postoperative RBC transfusions showed a decreasing trend in the crystalloid group; however, the difference was not statistically significant. (Fig. 2C, MD: -0.16U; 95%CI: -0.45, 0.14U; *P* = 0.30).

#### Postoperative blood loss

Postoperative blood loss or chest tube drainage in the first 24 h after surgery comparing albumin and crystalloid groups was reported in only three studies [23, 24, 30]. Meta-analysis of the pooled results showed no significant difference in postoperative blood loss (Fig. 2D, MD: 12.59 ml; 95%CI: -314.25, 339.43 ml; *P* = 0.94).

### Network meta-analysis

Due to the unavailability of data extracted from the included studies to facilitate an indirect analysis of the overall perioperative RBC transfusions, we conducted NMA for intraoperative and postoperative (the first 24 h) RBC transfusions. Postoperative blood loss was also observed in the NMA group. The details for above outcomes of the included studies on NMA is shown in Supplemental Table 4.

**Fig. 3:**
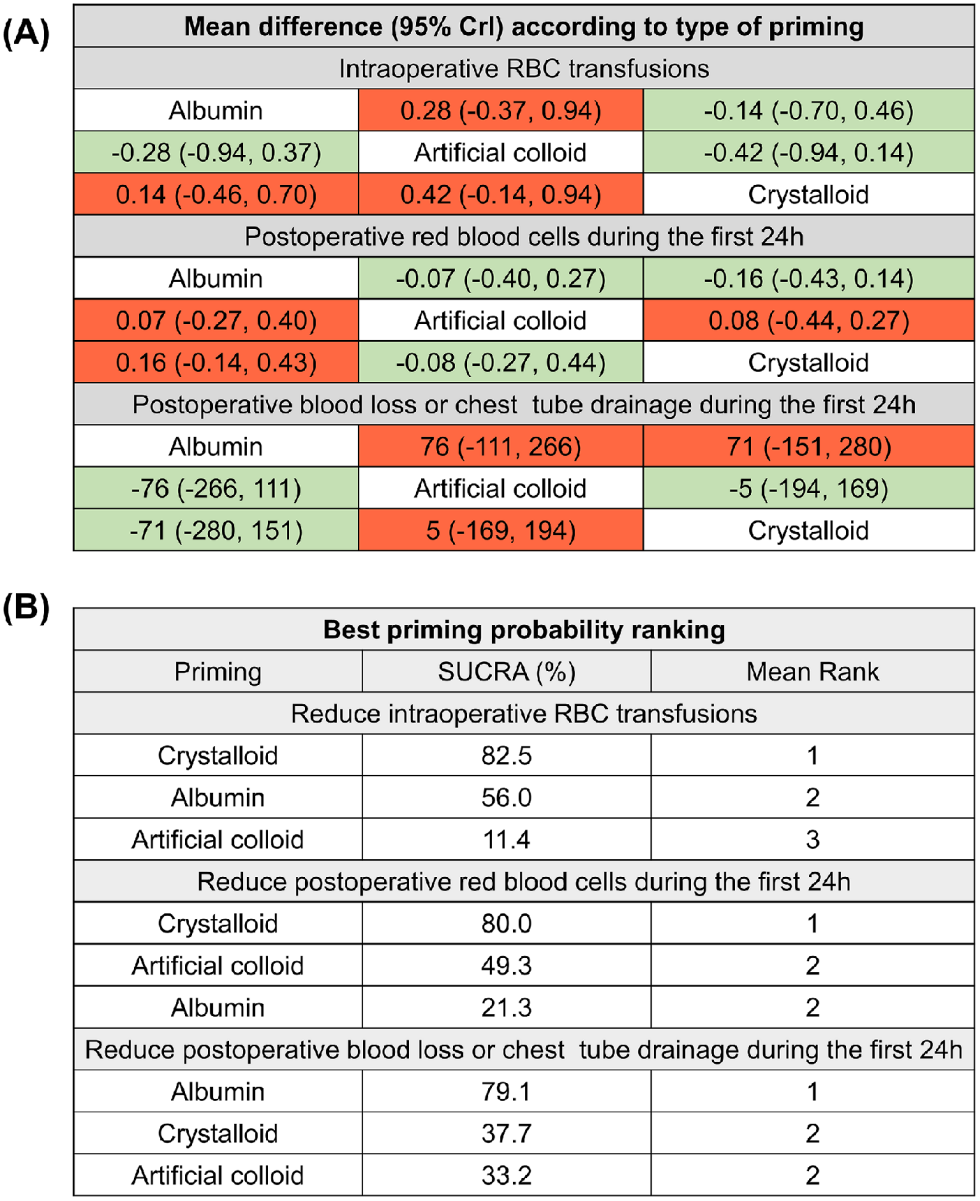
Tables with respect to NMA results. (**A**) Green color indicates favorable and red unfavorable mean difference with respective 95% credible interval. (**B**) The Surface Under the Cumulative Ranking (SUCRA) values represent the cumulative probability for each priming to being the best reduce RBC transfusions and blood loss (the closer the value is to 100%, the higher the likelihood that a priming is in the top rank). NMA, network meta-analysis

NMA analysis showed decreasing intraoperative and postoperative RBC transfusions and increasing postoperative blood loss trends in the crystalloid group compared with those in the albumin group (Fig. 3A). Because the 95% credible intervals all contain 0, the interpretability of the results is limited.

Furthermore, probability rank analysis was conducted. Based on the SUCRA values, the crystalloid priming strategy had a higher probability of reducing intraoperative and postoperative RBC transfusions but a lower probability of reducing postoperative blood loss compared with the albumin priming strategy (Fig. 3B).

### Risk of bias

**Fig. 4:**
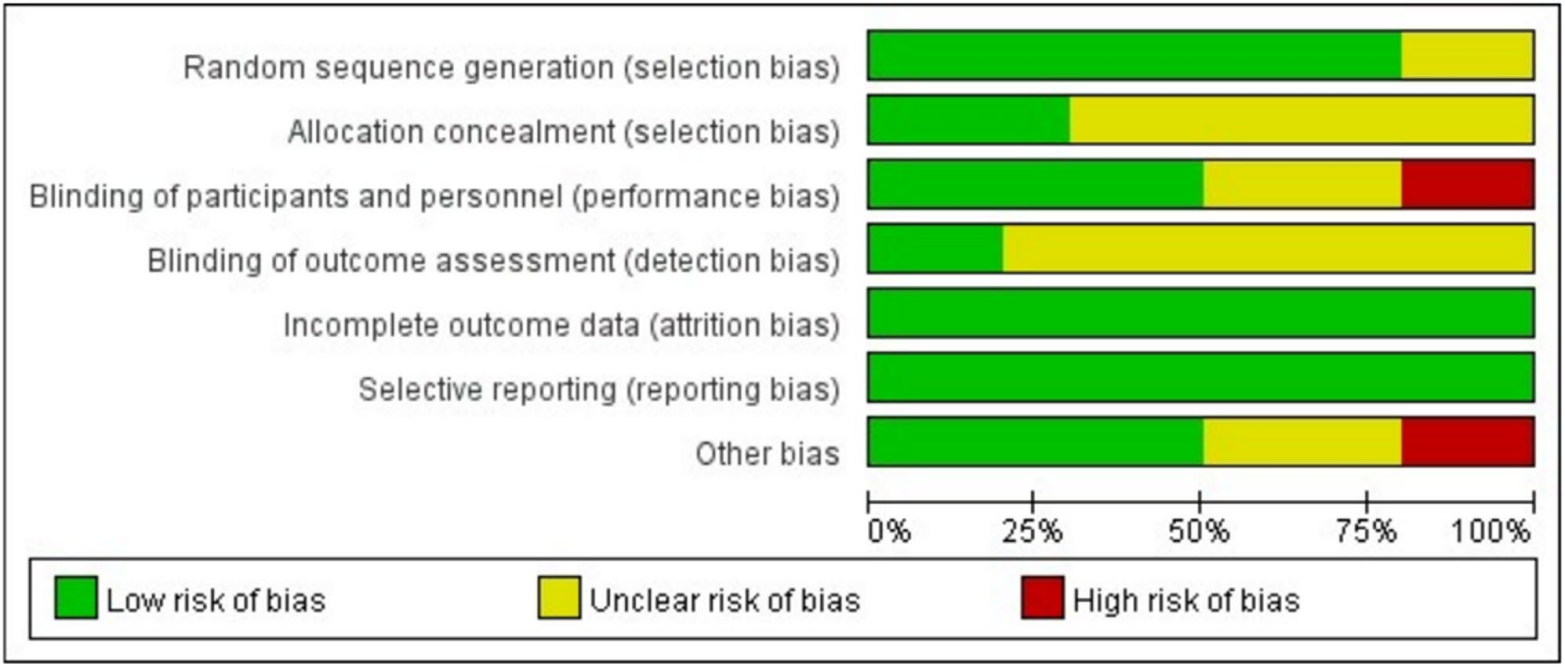
Risk of bias graph

The final risk of bias graph is shown in Fig. 4. The bias in each included study is summarized in Supplemental Fig. 2.

#### Selection bias

The standard randomization method was described in 8 studies. The correct allocation concealment was achieved in 2 studies [12, 24].

#### Performance bias

Proper double-blinding of the participants and personnel was achieved in 55.6% of the included RCTs. Three studies that not blinded the perfusionist [22, 23, 26], were considered to have unclear risk of bias. Two studies did not blind nurses, anesthesiologists, and surgeons [27, 30], which was considered to have a high risk of bias.

#### Detection bias

Adequate blinding of the outcome assessment was ensured in only 2 studies [12, 28] and was not mentioned in 7 studies.

#### Attrition bias

All studies reported reasons for dropouts. Thus, the attrition bias was considered low in all studies.

#### Other potential bias

Priming volume was not reported in 2 studies [22, 28] and the perioperative RBC transfusion threshold was not mentioned in 2 studies [12, 30]. Therefore, the unclear risk of other potential biases was considered in these 3 studies. In 1 study, tranexamic acid was used, which may affect the pre-specified outcomes (RBC transfusions and blood loss) and can be considered a high risk of bias. Owing to the low number of included studies, a funnel plot to assess publication bias was not conducted.

#### Reporting bias

Funnel plots for NMA analysis are provided in Supplementary Fig. 3 and did not suggest small study bias.

### Transitivity, heterogeneity, consistency and sensitivity analysis

We analyzed the distribution of baseline variables in the included studies between different priming strategies to assess transitivity. In either comparison, the difference between baseline variables was small (Supplemental Table 5). Supplemental Fig. 3 shows the results of the heterogeneity analysis. Supplemental Fig. 4 shows the results of the node-split consistency analysis. The P-values of all direct and indirect comparisons were greater than 0.05, indicating good consistency.

The leave-one-out sensitivity analysis results are displayed in Supplemental Table 6. There was no variation in the NMA analysis of intraoperative and postoperative (the first 24 h) RBC transfusions after changing study inputs. However, excluding Scott et al. changed the final SUCRA rank between albumin and crystalloid in postoperative blood loss (albumin had a lower SUCRA value than crystalloid). This suggests high heterogeneity among the included articles for meta-analysis.

### Meta-regression analysis

Supplementary Fig. 6 presents the results of the meta-regression analysis. Six important study-level factors (sample size, age, surgery type, male proportion, CPB time, and aortic cross-clamping time) were included for meta-regression analysis to explore the potential heterogeneity sources of NMA results. The results of meta-regression revealed that the inclusion of the above variables has no significant effect on the final NMA outcomes.

### Others

In addition, we analyzed the main postoperative factors in the included studies. There were no significant differences in ventilation time, ICU stay, hospital stay, AKI rate, and mortality among the three priming strategies. However, albumin priming strategy existed lower postoperative Hb counts than crystalloid (Supplemental Table 7, MD: -1.05 g/dl; 95%CI: -1.35, -0.74 g/dl; *P* < 0.01).

The certainty of evidence assessment according to the GRADE method is presented in Table 2.

**Table 2:** Certainty of evidence assessment

Crystalloid vs. Albumin							
Outcome	Direct evidence		Indirect evidence (Back-calculated)			Network meta-analysis	
	Mean Difference (95% confidence interval)	Quality		Mean Difference (95% credible interval)	Quality	Mean Difference (95% credible interval)	Quality
**Intraoperative RBC transfusions**	-0.20 [-0.39, -0.01]	Moderate†		0.11 [-0.90, 1.10]	Low^*¶^	-0.14 [-0.70, 0.46]	Low
**Postoperative RBC transfusions**	-0.16 [-0.45, 0.14]	Moderate†		0.25 [-1.10, 1.60]	Low^*¶^	-0.16 [-0.43, 0.14]	Low
**Postoperative blood loss**	12.59 [-314.25, 339.43]	Low^†‡¶^		132 [-290, 560]	Low^**‡¶^	71 [-151, 280]	Low
**Total perioperative RBC transfusions**	-0.68 [-1.26, -0.09]	High		-	-	-	-

^¶^Limitations (risk of bias). ^†^Inconsistency. ^‡^Imprecision. ^*^Contributing direct evidence of moderate quality. ^**^Contributing direct evidence of low or very low quality

## Discussion

In this study, we aimed to evaluate the safety of albumin and crystalloid priming strategies from a blood management perspective. The direct meta-analysis revealed that albumin priming strategy can increase perioperative RBC transfusions. After conducting NAM incorporating indirect analysis, albumin still displayed an increasing trend in RBC transfusions compared to crystalloid solutions. Additionally, we did not explore any differences in postoperative blood loss between albumin and crystalloid priming strategies in this study.

The main potential strength of albumin is that it can interact with vascular endothelial cells to maintain the integrity of the endothelial glycocalyx layer (EGL) [31]. EGL can be considered a dynamic layer between the vascular endothelial cell and the blood, capable of accommodating a significant amount of non-circulating plasma volume, providing 60% of the intravascular colloid osmotic pressure (COP) in the body [3]. During CPB, mechanical blood perfusion can lead to poor microcirculation and increased shear forces on local blood flow [32, 33]. Complete EGL can improve microcirculation by transmitting shear force signals, and its barrier function can cushion contact between blood cells and the endothelium [34, 35].

Previous clinical evidence suggests that adding albumin to the CPB priming solution can effectively preserve COP and reduce overall perioperative fluid transfusion, avoiding concomitant edema that can compromise organ function [36, 37]. However, the albumin priming strategy did not demonstrate an advantage in hemostasis effectiveness and even showed a detrimental effect. In a previous RCT comparing albumin, hydroxyethyl starch, and crystalloid solutions in 240 patients undergoing cardiac surgery with CPB, patients in the albumin group received RBC transfusions most frequently [24]. A recent randomized, double-blind, single-center clinical trial (The Albumin in Cardiac Surgery trial; ALBICS) involving 1386 patients undergoing on-pump cardiac surgery compared the safety of 4% albumin and Ringer acetate in CPB priming and intravenous volume replacement perioperatively. The results indicated that the albumin group received more RBC transfusions and had clinically significant bleeding [38]. In our study, these two RCTs were also the primary sources of the direct meta-analysis, thus yielding results demonstrating the association between the albumin priming strategy and an increase in RBC transfusions. Considering the limited amount of direct evidence, we conducted a NMA to expand the sample pool. The NMA results still indicated a higher RBC transfusion with albumin priming than with crystalloids, but there was no statistically significant difference. It should be noted that our study exhibited large differences in quality of evidence between the direct and indirect evidence. Although there are few studies that provide direct evidence, the sample size of these studies is large and the research quality is high. In contrast, RCTs that provide indirect evidence are mostly old and have a small sample size, which results in low-quality evidence. Therefore, despite the lack of statistical difference in NMA results, our study still supports that the albumin priming strategy could increase perioperative RBC transfusion compared to crystalloid solutions.

Previous animal experiments have provided evidence that endogenous albumin may exert concentration-dependent anticoagulant effects [39]. Moreover, in previous clinical studies, patients in the albumin priming group had lower Hb concentrations after surgery, which was also observed in this study [12, 23, 24]. The more profound hemodilution effect of albumin, leading to an earlier reduction in Hb, might explain the increased RBC receiving albumin administration [24]. However, excessive hemodilution of albumin may not have serious consequences on postoperative blood loss. In addition, the final concentration of albumin in crystalloid solution is also an important consideration. A recent network meta-analysis revealed that the use of iso-oncotic HA (4-5% final concentration) was associated with the lowest blood loss in the first 24 h after surgery compared with other priming strategies. In contrast, hyper-oncotic HA (25% final concentration) resulted in the longest hospital stay and CPB time compared to other priming strategies, suggesting the poor effect of hyper-oncotic HA [40]. In our meta-analysis, all included albumin priming strategies were isotonic. Our NMA evidence also showed that albumin exist high probability of reducing postoperative blood loss. Even so, it is still imperative to evaluate the suitability of the albumin priming strategies in specific populations. Additional intraoperative albumin supplementation may be necessary in patients with hypoalbuminemia since perioperative hypoalbuminemia has been proven to be independently associated with increased morbidity and mortality after cardiac operations [41, 42]. Furthermore, the infusion volume of the experimental fluid was also a key point. It is interesting that the two RCTs, which provided the most direct evidence in our study, conducted the entire perioperative fluid administration comparison (priming and perioperative volume replacement) [12, 24] thus, we speculate that the impact of albumin on hemostasis is related to the transfusion volume in cardiac surgery. In other words, more studies are needed to evaluate the hemostasis between albumin and crystalloid solutions when considering the priming solution alone or even applying a minimally invasive extracorporeal circulation (MiECC) system to reduce the priming volume.

### Limitations

This study has some limitations. First, in the process of article screening and data extraction, to minimize heterogeneity, we defined the postoperative time as within 24 h and standardized the RBC measurements, leading to the exclusion of many studies. However, this ensured that the studies ultimately included in the analysis had a lower potential bias and higher credibility of the obtained results. Second, the varieties of priming strategies among different centers, including the types of crystals or artificial colloids, concentration of albumin, and priming volume, would be uncontrollable factors in this analysis. Third, the included studies were not consistent in perioperative blood management, especially in terms of the different thresholds for perioperative RBC transfusions. This inconsistency may also have lowered the quality of the evidence in this study. Fourth, this study did not differentiate between artificial colloids, such as gelatin and HES, in the analysis. Owing to concerns about the nephrotoxicity of HES, many centers tend to prefer gelatin as a colloid preloading solution [43, 44]. However, there is currently no clear evidence indicating a significant difference in hemostatic effectiveness between gelatin and HES preloading strategies [45, 46], but combining different types of artificial colloids could potentially affect research evidence.

### Strength

Despite above limitations, the whole report adhered rigorously to the protocol registered with PROSPERO, and all pre-defined outcomes were carefully analyzed. As the first NMA study to compare the impact of RBC transfusions between albumin and crystalloid priming strategies, the results of this study would be of significant value in optimizing blood preservation measures in patients undergoing cardiovascular surgery with CPB.

## Conclusion

Direct evidence has revealed that the albumin priming strategy has a significant effect on perioperative RBC transfusion. After the inclusion of indirect evidence, the NMA results further indicated that the albumin priming strategy resulted in more perioperative RBC receiving than crystalloids, although the statistical significance was limited. More consideration should be given to the application of the albumin priming strategy in on-pump cardiac surgery. Future research should focus on the safety and effectiveness of albumin and crystalloid priming strategies in specific populations (such as patients with hypoalbuminemia) or in the MiECC system.

### Electronic supplementary material

Below is the link to the electronic supplementary material.

**Supplementary Material 1**: Supplemental Figure 1. Search strategy.

**Supplementary Material 2**: Supplemental Figure 2. Summary of the risk of bias among the randomized controlled trials using the Cochrane Risk of Bias tool.

**Supplementary Material 3**: Supplemental Figure 3. Reporting bias funnel plots for network meta-analysis.

**Supplementary Material 4**: Supplemental Figure 4. Analysis of heterogeneity.

**Supplementary Material 5**: Supplemental Figure 5. Node-splitting analysis of inconsistency.

**Supplementary Material 6**: Supplemental Figure 6. Meta-regression for network meta-analysis.

**Supplementary Material 7**: Supplemental Table 1. PRISMA (Preferred Reporting Items for Systematic review and Meta-Analysis) 2020 checklist: an updated guideline for reporting systematic reviews.

**Supplementary Material 8**: Supplemental Table 2. PICOS criteria for the network meta-analysis.

**Supplementary Material 9**: Supplemental Table 3. Articles excluded after full-text reading.

**Supplementary Material 10**: Supplemental Table 4. Details for pre-defined outcomes of studies included for network meta-analysis.

**Supplementary Material 11**: Supplemental Table 5. Baseline variables of the included studies.

**Supplementary Material 12**: Supplemental Table 6. Leave-one-out sensitivity analysis for network meta-analysis.

**Supplementary Material 13**: Supplemental Table 7. Main postoperative variables of the included studies.

## Data Availability

No datasets were generated or analysed during the current study.

## Abbreviations

CPB: cardiopulmonary bypass
HES: hydroxyethyl starch
RBC: red blood cell
RCT: randomized controlled trial
NMA: network meta-analysis
ICU: intensive care unit
AKI: acute kidney injury
MD: mean differences
CI: confidence intervals
Crl: credible interval
SUCRA: surface under the cumulative ranking values
GRADE: Grading of Recommendations Assessment, Development and Evaluation
EGL: glycocalyx layer
COP: colloid osmotic pressure
MiECC: minimally invasive extracorporeal circulation
