# Supplementary figures and images for: Effects of albumin and crystalloid priming strategies on red blood cell transfusions in on-pump cardiac surgery: a network meta-analysis
Source: BMC Anesthesiol. 2024 Jan 16;24:26. doi: 10.1186/s12871-024-02414-y (PMC10790517; doi:10.1186/s12871-024-02414-y)

**
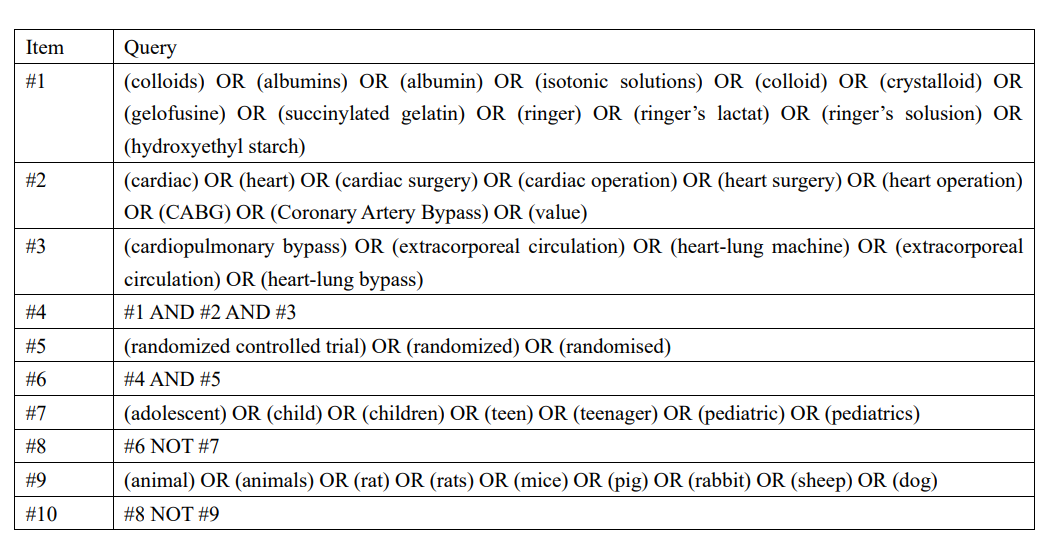
**

**Supplemental Figure 1.** Search strategy.

Supplement: Supplementary file 1 — Supplementary Material 1: Supplemental Figure 1. Search strategy. [file 12871_2024_2414_MOESM1_ESM.docx]
