# Supplementary material for: Effects of albumin and crystalloid priming strategies on red blood cell transfusions in on-pump cardiac surgery: a network meta-analysis
Source: BMC Anesthesiol. 2024 Jan 16;24:26. doi: 10.1186/s12871-024-02414-y (PMC10790517; doi:10.1186/s12871-024-02414-y)
Supplement: Supplementary file 2 — Supplementary Material 2: Supplemental Figure 2. Summary of the risk of bias among the randomized controlled trials using the Cochrane Risk of Bias tool. [file 12871_2024_2414_MOESM2_ESM.docx]

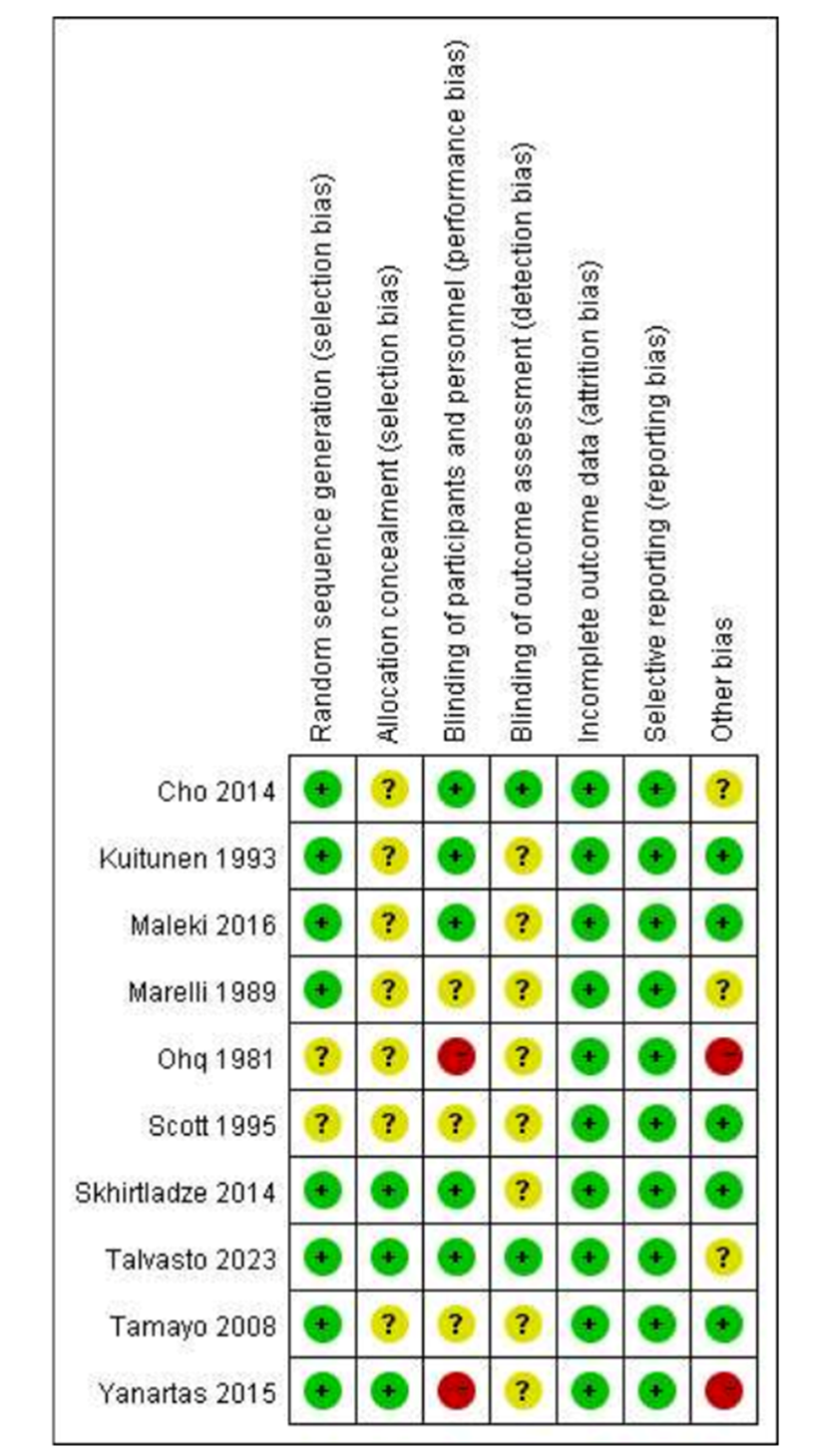


**Supplemental Figure 2.** Summary of the risk of bias among the randomized controlled trials using the Cochrane Risk of Bias tool.
