# Supplementary material for: Effects of albumin and crystalloid priming strategies on red blood cell transfusions in on-pump cardiac surgery: a network meta-analysis
Source: BMC Anesthesiol. 2024 Jan 16;24:26. doi: 10.1186/s12871-024-02414-y (PMC10790517; doi:10.1186/s12871-024-02414-y)
Supplement: Supplementary file 3 — Supplementary Material 3: Supplemental Figure 3. Reporting bias funnel plots for network meta-analysis. [file 12871_2024_2414_MOESM3_ESM.docx]

**
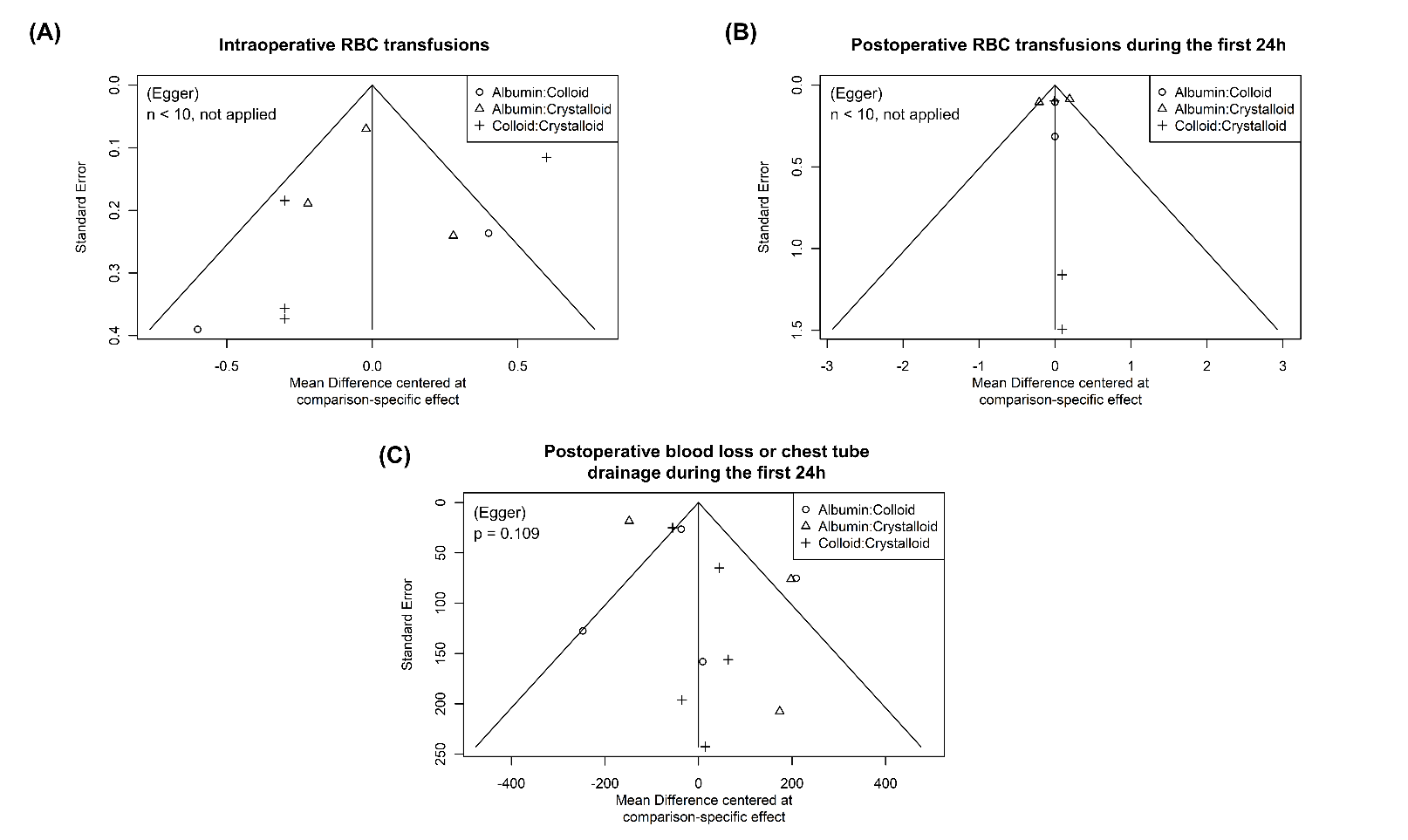
**

**Supplemental Figure 3.** Reporting bias funnel plots for network meta-analysis. (A) Intraoperative red blood cells transfusions. (B) Postoperative red blood cells transfusions during the first 24h. (C) Postoperative blood loss or chest tube drainage during the first 24h. NMA, network meta-analysis.
