# Supplementary material for: Effects of albumin and crystalloid priming strategies on red blood cell transfusions in on-pump cardiac surgery: a network meta-analysis
Source: BMC Anesthesiol. 2024 Jan 16;24:26. doi: 10.1186/s12871-024-02414-y (PMC10790517; doi:10.1186/s12871-024-02414-y)
Supplement: Supplementary file 5 — Supplementary Material 5: Supplemental Figure 5. Node-splitting analysis of inconsistency. [file 12871_2024_2414_MOESM5_ESM.docx]

**
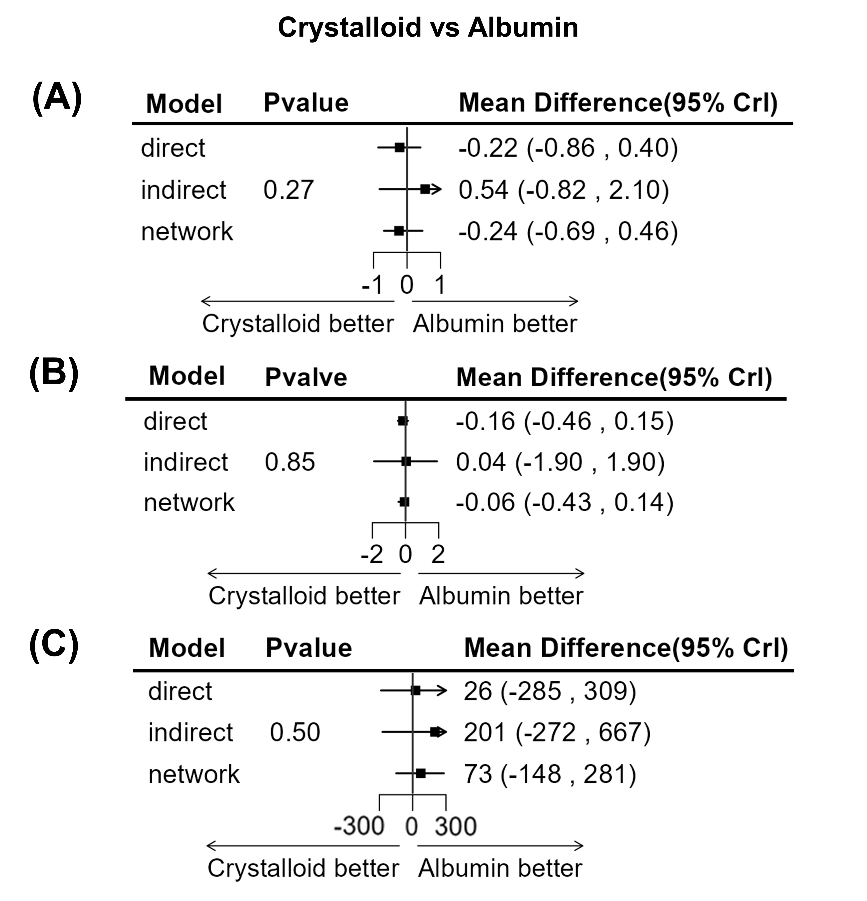
**

**Supplemental Figure 5.** Node-splitting analysis of inconsistency. (A) Intraoperative red blood cells transfusions. (B) Postoperative red blood cells transfusions during the first 24h. (C) Postoperative blood loss or chest tube drainage during the first 24h. Crl, Credible Interval.
