# Supplementary material for: Effects of albumin and crystalloid priming strategies on red blood cell transfusions in on-pump cardiac surgery: a network meta-analysis
Source: BMC Anesthesiol. 2024 Jan 16;24:26. doi: 10.1186/s12871-024-02414-y (PMC10790517; doi:10.1186/s12871-024-02414-y)
Supplement: Supplementary file 6 — Supplementary Material 6: Supplemental Figure 6. Meta-regression for network meta-analysis. [file 12871_2024_2414_MOESM6_ESM.docx]

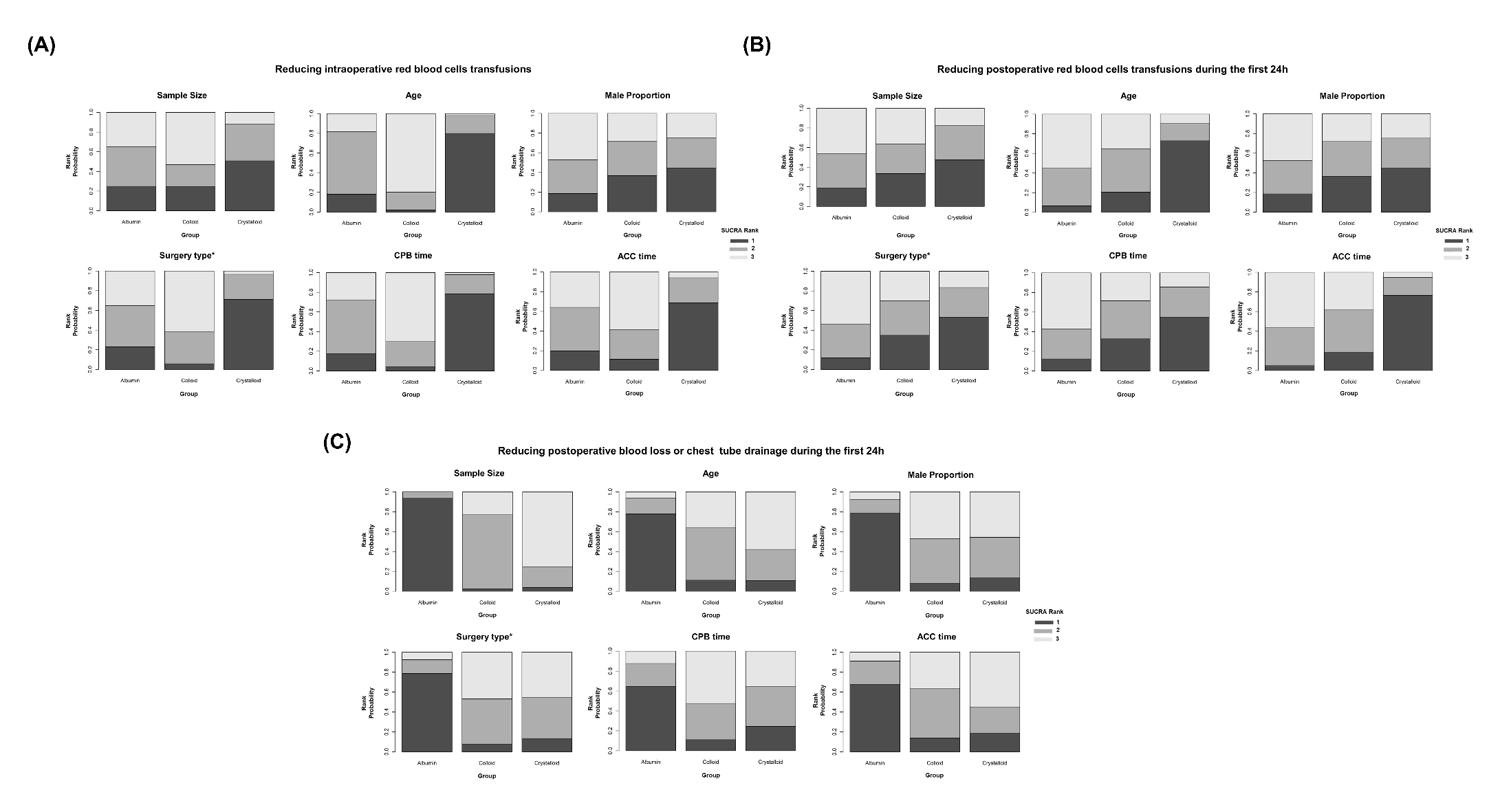


**Supplemental Figure 6.** Meta-regression for network meta-analysis. Including six important study-level factors (sample size, age, surgery type, male proportion, CPB time and aortic cross-clamping time) respectively to conduct meta-regression for network meta-analysis. The final results were presented as the SUCRA rank probability plots. The outcomes evaluated were: (A) Reducing intraoperative red blood cells transfusions. (B) Reducing postoperative red blood cells transfusions during the first 24h. (C) Reducing postoperative blood loss or chest tube drainage during the first 24h. *The surgery types prescribed in the studies: 1. Valve; 2. CABG; 3. Valve or CABG; 4. Valve, CABG or Aortic. CPB, cardiopulmonary bypass; ACC, aortic cross-clamping.
