# Supplementary material for: Effects of albumin and crystalloid priming strategies on red blood cell transfusions in on-pump cardiac surgery: a network meta-analysis
Source: BMC Anesthesiol. 2024 Jan 16;24:26. doi: 10.1186/s12871-024-02414-y (PMC10790517; doi:10.1186/s12871-024-02414-y)
Supplement: Supplementary file 8 — Supplementary Material 8: Supplemental Table 2. PICOS criteria for the network meta-analysis. [file 12871_2024_2414_MOESM8_ESM.docx]

**Supplemental Table 2.** PICOS criteria for network meta-analysis.

| **Criteria** | **Explain** |
| --- | --- |
| P (Population) | Adult patients undergoing cardiovascular surgery with CPB |
| I (Intervention) | Human albumin priming strategy |
| C (Comparison) | Crystalloid priming strategy |
| O (Outcome) | Primary outcomes: RBC transfusion volume in units at 24 hours.  Secondary outcomes: postoperative blood loss (the first 24 hours). |
| S (Study design) | Randomized controlled trials |
