# Supplementary material for: Effects of albumin and crystalloid priming strategies on red blood cell transfusions in on-pump cardiac surgery: a network meta-analysis
Source: BMC Anesthesiol. 2024 Jan 16;24:26. doi: 10.1186/s12871-024-02414-y (PMC10790517; doi:10.1186/s12871-024-02414-y)
Supplement: Supplementary file 9 — Supplementary Material 9: Supplemental Table 3. Articles excluded after full-text reading. [file 12871_2024_2414_MOESM9_ESM.docx]

**Supplemental Table 3.** Articles excluded after full-text reading.

| **Excluded reasons** | **Number** |
| --- | --- |
| Not included primary or secondary endpoints [1-11]. | 11 |
| Nor measure RBC by unit [12, 13]. | 2 |
| Data missing standard deviation or interquartile range [14-17]. | 4 |
| Not measure the postoperative RBC transfusions in the first 24h [18-22]. | 5 |

**Reference:**

1. Kamada T, McMillan DE, Sternlieb JJ, Bjork VO, Otsuji S: **Albumin prevents erythrocyte crenation in patients undergoing extracorporeal circulation**. *Scand J Thorac Cardiovasc Surg* 1988, **22**(2):155-158.

2. Himpe D, Van Cauwelaert P, Neels H, Stinkens D, Van den Fonteyne F, Theunissen W, Muylaert P, Hermans C, Goossens G, Moeskops J *et al*: **Priming solutions for cardiopulmonary bypass: comparison of three colloids**. *J Cardiothorac Vasc Anesth* 1991, **5**(5):457-466.

3. Hoeft A, Korb H, Mehlhorn U, Stephan H, Sonntag H: **Priming of cardiopulmonary bypass with human albumin or Ringer lactate: effect on colloid osmotic pressure and extravascular lung water**. *Br J Anaesth* 1991, **66**(1):73-80.

4. Jenkins IR, Curtis AP: **The combination of mannitol and albumin in the priming solution reduces positive intraoperative fluid balance during cardiopulmonary bypass**. *Perfusion* 1995, **10**(5):301-305.

5. Tølløfsrud S, Svennevig JL, Breivik H, Kongsgaard U, Ozer M, Hysing E, Mohr B, Seem E, Geiran O, Abdelnour M *et al*: **Fluid balance and pulmonary functions during and after coronary artery bypass surgery: Ringer's acetate compared with dextran, polygeline, or albumin**. *Acta Anaesthesiol Scand* 1995, **39**(5):671-677.

6. Buhre W, Hoeft A, Schorn B, Weyland A, Scholz M, Sonntag H: **Acute affect of mitral calve replacement on extravascular lung water in patients receiving colloid or crystalloid priming of cardiopulmonary bypass**. *Br J Anaesth* 1997, **79**(3):311-316.

7. Tigchelaar I, Gallandat Huet RC, Korsten J, Boonstra PW, van Oeveren W: **Hemostatic effects of three colloid plasma substitutes for priming solution in cardiopulmonary bypass**. *Eur J Cardiothorac Surg* 1997, **11**(4):626-632.

8. Boks RH, van Herwerden LA, Takkenberg JJ, van Oeveren W, Gu YJ, Wijers MJ, Bogers AJ: **Is the use of albumin in colloid prime solution of cardiopulmonary bypass circuit justified?** *Ann Thorac Surg* 2001, **72**(3):850-853.

9. Boks RH, Wijers MJ, Hofland J, Takkenberg JJ, Bogers AJ: **Low molecular starch versus gelatin plasma expander during CPB: does it make a difference?** *Perfusion* 2007, **22**(5):333-337.

10. Kamra C, Beney A: **Human albumin in extracorporeal prime: effect on platelet function and bleeding**. *Perfusion* 2013, **28**(6):536-540.

11. Isoda S, Izubuchi R, Yamazaki I, Nakayama Y, Yano Y, Masuda M: **Priming and replenishment in cardiopulmonary bypass with hydroxyethyl starch 130/0.4 decreases fluid overbalance without renal dysfunction or bleeding in adult valve surgery**. *Gen Thorac Cardiovasc Surg* 2019, **67**(4):374-376.

12. Ohqvist G, Settergren G, Lundberg S: **Pulmonary oxygenation, central haemodynamics and glomerular filtration following cardiopulmonary bypass with colloid or non-colloid priming solution**. *Scand J Thorac Cardiovasc Surg* 1981, **15**(3):257-262.

13. Sade RM, Stroud MR, Crawford FA, Jr., Kratz JM, Dearing JP, Bartles DM: **A prospective randomized study of hydroxyethyl starch, albumin, and lactated Ringer's solution as priming fluid for cardiopulmonary bypass**. *J Thorac Cardiovasc Surg* 1985, **89**(5):713-722.

14. Zarro DL, Palanzo DA, Phillips TG: **Albumin in the pump prime: its effect on postoperative weight gain**. *Perfusion* 2001, **16**(2):129-135.

15. Boldt J, Brosch C, Ducke M, Papsdorf M, Lehmann A: **Influence of volume therapy with a modern hydroxyethylstarch preparation on kidney function in cardiac surgery patients with compromised renal function: a comparison with human albumin**. *Crit Care Med* 2007, **35**(12):2740-2746.

16. Tiryakioğlu O, Yildiz G, Vural H, Goncu T, Ozyazicioglu A, Yavuz S: **Hydroxyethyl starch versus Ringer solution in cardiopulmonary bypass prime solutions (a randomized controlled trial)**. *J Cardiothorac Surg* 2008, **3**:45.

17. Boldt J, Suttner S, Brosch C, Lehmann A, Röhm K, Mengistu A: **Cardiopulmonary bypass priming using a high dose of a balanced hydroxyethyl starch versus an albumin-based priming strategy**. *Anesth Analg* 2009, **109**(6):1752-1762.

18. Jansen PG, te Velthuis H, Wildevuur WR, Huybregts MA, Bulder ER, van der Spoel HI, Sturk A, Eijsman L, Wildevuur CR: **Cardiopulmonary bypass with modified fluid gelatin and heparin-coated circuits**. *Br J Anaesth* 1996, **76**(1):13-19.

19. Choi YS, Shim JK, Hong SW, Kim JC, Kwak YL: **Comparing the effects of 5% albumin and 6% hydroxyethyl starch 130/0.4 on coagulation and inflammatory response when used as priming solutions for cardiopulmonary bypass**. *Minerva Anestesiol* 2010, **76**(8):584-591.

20. Gurbuz HA, Durukan AB, Salman N, Tavlasoglu M, Durukan E, Ucar HI, Yorgancioglu C: **Hydroxyethyl starch 6%, 130/0.4 vs. a balanced crystalloid solution in cardiopulmonary bypass priming: a randomized, prospective study**. *J Cardiothorac Surg* 2013, **8**:71.

21. Schramko A, Suojaranta-Ylinen R, Niemi T, Pesonen E, Kuitunen A, Raivio P, Salmenperä M: **The use of balanced HES 130/0.42 during complex cardiac surgery; effect on blood coagulation and fluid balance: a randomized controlled trial**. *Perfusion* 2015, **30**(3):224-232.

22. Svendsen Ø S, Farstad M, Mongstad A, Haaverstad R, Husby P, Kvalheim VL: **Is the use of hydroxyethyl starch as priming solution during cardiac surgery advisable? A randomized, single-center trial**. *Perfusion* 2018, **33**(6):483-489.
