# Supplementary material for: Effects of albumin and crystalloid priming strategies on red blood cell transfusions in on-pump cardiac surgery: a network meta-analysis
Source: BMC Anesthesiol. 2024 Jan 16;24:26. doi: 10.1186/s12871-024-02414-y (PMC10790517; doi:10.1186/s12871-024-02414-y)
Supplement: Supplementary file 10 — Supplementary Material 10: Supplemental Table 4. Details for pre-defined outcomes of studies included for network meta-analysis. [file 12871_2024_2414_MOESM10_ESM.docx]

**Supplemental Table 4. Details for pre-defined outcomes of studies included for network meta-analysis.**

| **Studies** | **Type** | **Number** | **Mean** | **Standard deviation** |
| --- | --- | --- | --- | --- |
| **Intraoperative RBC transfusions** | |  |  |  |
| Marelli 1989[1] | Albumin | 51 | 3 | 1.3 |
|  | Crystalloid | 49 | 2.5 | 1.1 |
| Kuitunen 1993[2] | Artificial colloid | 15 | 1.4 | 0.4 |
|  | Crystalloid | 15 | 0.5 | 0.2 |
| Cho 2014[3] | Albumin | 18 | 0 | 0.74 |
|  | Artificial colloid | 18 | 1 | 1.48 |
| Skhirtladze 2014[4] | Albumin | 76 | 0 | 1.48 |
|  | Artificial colloid | 81 | 0 | 1.48 |
|  | Crystalloid | 79 | 0 | 0.74 |
| Yanartas 2015[5] | Artificial colloid | 35/34 | 0/0 | 1.48/1.48 |
|  | Crystalloid | 31/32 | 1/1 | 1.48/1.48 |
| Talvasto 2023[6] | Albumin | 693 | 0.8 | 1.3 |
|  | Crystalloid | 693 | 0.6 | 1.3 |
| **Postoperative RBC transfusions** | |  |  |  |
| Cho 2014[3] | Albumin | 18 | 0 | 0.74 |
|  | Artificial colloid | 18 | 0 | 1.11 |
| Skhirtladze 2014[4] | Albumin | 76 | 0 | 0.68 |
|  | Artificial colloid | 81 | 0 | 0.617 |
|  | Crystalloid | 79 | 0 | 0.617 |
| Yanartas 2015[5] | Artificial colloid | 35/34 | 1/1 | 5.18/4.44 |
|  | Crystalloid | 31/32 | 1/1 | 5.93/5.93 |
| Talvasto 2023[6] | Albumin | 693 | 0.5 | 1.2 |
|  | Crystalloid | 693 | 0.2 | 1.9 |
| **Postoperative blood loss** | |  |  |  |
| Ohqvist 1981[7] | Albumin | 7 | 2287 | 2259 |
|  | Crystalloid | 7 | 2107 | 2200 |
| Scott 1995[8]; | Albumin | 32 | 843 | 81 |
|  | Artificial colloid | 29 | 954 | 120 |
|  | Crystalloid | 32 | 1024 | 65 |
| Tamayo 2008[9]; | Artificial colloid | 22 | 995 | 496.6 |
|  | Crystalloid | 22 | 946 | 537.9 |
| Cho 2014[3]; | Albumin | 18 | 430 | 300 |
|  | Artificial colloid | 18 | 495 | 600 |
| Skhirtladze 2014[4]; | Albumin | 76 | 835 | 524.4 |
|  | Artificial colloid | 81 | 700 | 407.4 |
|  | Crystalloid | 79 | 670 | 414.8 |
| Yanartas 2015[5]; | Artificial colloid | 35/34 | 300/350 | 814.8/814.8 |
|  | Crystalloid | 31/32 | 400/400 | 963/963 |
| Maleki 2016[10]; | Albumin | 30 | 799.84 | 382.75 |
|  | Artificial colloid | 30 | 1121 | 584.58 |

**Reference:**

1. Marelli D, Paul A, Samson R, Edgell D, Angood P, Chiu RC: **Does the addition of albumin to the prime solution in cardiopulmonary bypass affect clinical outcome? A prospective randomized study**. *J Thorac Cardiovasc Surg* 1989, **98**(5 Pt 1):751-756.

2. Kuitunen A, Hynynen M, Salmenperä M, Heinonen J, Vahtera E, Verkkala K, Myllylä G: **Hydroxyethyl starch as a prime for cardiopulmonary bypass: effects of two different solutions on haemostasis**. *Acta Anaesthesiol Scand* 1993, **37**(7):652-658.

3. Cho JE, Shim JK, Song JW, Lee HW, Kim DH, Kwak YL: **Effect of 6% hydroxyethyl starch 130/0.4 as a priming solution on coagulation and inflammation following complex heart surgery**. *Yonsei Med J* 2014, **55**(3):625-634.

4. Skhirtladze K, Base EM, Lassnigg A, Kaider A, Linke S, Dworschak M, Hiesmayr MJ: **Comparison of the effects of albumin 5%, hydroxyethyl starch 130/0.4 6%, and Ringer's lactate on blood loss and coagulation after cardiac surgery**. *Br J Anaesth* 2014, **112**(2):255-264.

5. Yanartas M, Baysal A, Aydın C, Ay Y, Kara I, Aydın E, Cevirme D, Köksal C, Sunar H: **The effects of tranexamic acid and 6% hydroxyethyl starch (HES) solution (130/0.4) on postoperative bleeding in coronary artery bypass graft (CABG) surgery**. *Int J Clin Exp Med* 2015, **8**(4):5959-5971.

6. Talvasto A, Ilmakunnas M, Raivio P, Vlasov H, Hiippala S, Suojaranta R, Wilkman E, Petaja L, Helve O, Juvonen T *et al*: **Albumin Infusion and Blood Loss After Cardiac Surgery**. *Ann Thorac Surg* 2023, **116**(2):392-399.

7. Ohqvist G, Settergren G, Bergstrom K, Lundberg S: **Plasma colloid osmotic pressure during open-heart surgery using non-colloid or colloid priming solution in the extracorporeal circuit**. *Scand J Thorac Cardiovasc Surg* 1981, **15**(3):251-255.

8. Scott DA, Hore PJ, Cannata J, Masson K, Treagus B, Mullaly J: **A comparison of albumin, polygeline and crystalloid priming solutions for cardiopulmonary bypass in patients having coronary artery bypass graft surgery**. *Perfusion* 1995, **10**(6):415-424.

9. Tamayo E, Alvarez FJ, Alonso O, Castrodeza J, Bustamante R, Gómez-Herreras JI, Florez S, Rodríguez R: **The inflammatory response to colloids and crystalloids used for pump priming during cardiopulmonary bypass**. *Acta Anaesthesiol Scand* 2008, **52**(9):1204-1212.

10. Hosseinzadeh Maleki M, Derakhshan P, Rahmanian Sharifabad A, Amouzeshi A: **Comparing the Effects of 5% Albumin and 6% Hydroxyethyl Starch 130/0.4 (Voluven) on Renal Function as Priming Solutions for Cardiopulmonary Bypass: A Randomized Double Blind Clinical Trial**. *Anesth Pain Med* 2016, **6**(1):e30326.
