# Supplementary material for: Effects of albumin and crystalloid priming strategies on red blood cell transfusions in on-pump cardiac surgery: a network meta-analysis
Source: BMC Anesthesiol. 2024 Jan 16;24:26. doi: 10.1186/s12871-024-02414-y (PMC10790517; doi:10.1186/s12871-024-02414-y)
Supplement: Supplementary file 11 — Supplementary Material 11: Supplemental Table 5. Baseline variables of the included studies. [file 12871_2024_2414_MOESM11_ESM.docx]

**Supplemental Table 5. Baseline variables of the included studies.**

| **Covariate** | **Number of trials reporting the variable (%)** | **Mean Difference or Odds Ratio (95% CI)** | **I^2^ (%)** | ***P*-value** |
| --- | --- | --- | --- | --- |
| **Albumin vs Crystalloid** |  |  |  |  |
| **Age (year)** | 5/5 (100%) | 1.44 [-1.00, 3.88] | 83 | 0.25 |
| **Male Sex** | 5/5 (100%) | 0.98 [0.77, 1.23] | 0 | 0.84 |
| **BMI (kg/m^2^)** | 2/5 (40%) | 0.35 [-0.12, 0.81] | 0 | 0.15 |
| **CPB time (min)** | 5/5 (100%) | -2.12 [-4.33, 0.10] | 0 | 0.06 |
| **ACC time (min)** | 3/5 (60%) | -2.46 [-4.14, -0.79] | 0 | <0.01 |
| **Preoperative Hct (%) or Hb (g/dl) *** | 3/5 (60%) | -0.04 [-0.14, 0.06] | 97 | 0.42 |
| **Albumin vs Artificial Colloid** |  |  |  |  |
| **Age (year)** | 4/4 (100%) | 2.91 [-0.68, 6.50] | 49 | 0.11 |
| **Male Sex** | 3/4 (75%) | 1.34 [0.80, 2.25] | 0 | 0.27 |
| **BMI (kg/m^2^)** | 2/4 (50%) | 0.24 [-1.35, 0.87] | 0 | 0.68 |
| **CPB time (min)** | 4/4 (100%) | -2.21 [-5.44, 1.01] | 7 | 0.18 |
| **ACC time (min)** | 4/4 (100%) | 7.04 [-6.18, 20.26] | 94 | 0.30 |
| **Preoperative Hct (%) or Hb (g/dl) *** | 3/4 (75%) | -0.35 [-0.8, 0.09] | 48 | 0.12 |
| **Artificial Colloid vs Crystalloid** |  |  |  |  |
| **Age (year)** | 5/5 (100%) | -0.47 [-1.88, 0.94] | 66 | 0.51 |
| **Male Sex** | 4/5 (80%) | 1.00 [0.57, 1.75] | 25 | 0.99 |
| **BMI (kg/m^2^)** | 1/5 (20%) | - | - | - |
| **CPB time (min)** | 5/5 (100%) | 0.40 [-2.01, 2.82] | 0 | 0.75 |
| **ACC time (min)** | 5/5 (100%) | -1.72 [-3.19, -0.27] | 0 | 0.02 |
| **Preoperative Hct (%) or Hb (g/dl) *** | 4/5 (80%) | -0.82 [-1.69, 0.06] | 91 | <0.01 |

Hct, Hematocrit; Hb, Hemoglobin; CPB, cardiopulmonary bypass; ACC, aortic cross-clamping. *If not including Hct, Hb instead, using standardized mean difference to compare characteristics
