# Supplementary material for: Effects of albumin and crystalloid priming strategies on red blood cell transfusions in on-pump cardiac surgery: a network meta-analysis
Source: BMC Anesthesiol. 2024 Jan 16;24:26. doi: 10.1186/s12871-024-02414-y (PMC10790517; doi:10.1186/s12871-024-02414-y)
Supplement: Supplementary file 12 — Supplementary Material 12: Supplemental Table 6. Leave-one-out sensitivity analysis for network meta-analysis. [file 12871_2024_2414_MOESM12_ESM.docx]

**Supplemental Table 6.** Leave-one-out sensitivity analysis for network meta-analysis.

| **Best priming probability ranking** | | | |
| --- | --- | --- | --- |
| **Excluded study** | **Priming** | **SUCRA (%)** | **Mean Rank** |
| **Reduce intraoperative RBC transfusions** | | | |
| **Marelli 1989** | Albumin | 66.7 | 2 |
|  | Colloid | 11.2 | 3 |
|  | Crystalloid | 72.1 | 1 |
| **Kuitunen 1993** | Albumin | 45.0 | 2 |
|  | Colloid | 27.1 | 3 |
|  | Crystalloid | 77.9 | 1 |
| **Cho 2014** | Albumin | 37.1 | 2 |
|  | Colloid | 26.1 | 3 |
|  | Crystalloid | 86.8 | 1 |
| **Skhirtladze 2014** | Albumin | 57.9 | 2 |
|  | Colloid | 9.3 | 3 |
|  | Crystalloid | 82.8 | 1 |
| **Yanartas 2015**  **(Group 1)** | Albumin | 56.0 | 2 |
|  | Colloid | 9.4 | 3 |
|  | Crystalloid | 84.6 | 1 |
| **Yanartas 2015**  **(Group 2)** | Albumin | 56.3 | 2 |
|  | Colloid | 9.4 | 3 |
|  | Crystalloid | 84.3 | 1 |
| **Talvasto 2023** | Albumin | 59.0 | 2 |
|  | Colloid | 14.2 | 3 |
|  | Crystalloid | 76.8 | 1 |
| **Reduce postoperative red blood cells during the first 24h** | | | |
| **Cho 2014** | Albumin | 21.7 | 3 |
|  | Colloid | 51.4 | 2 |
|  | Crystalloid | 76.9 | 1 |
| **Skhirtladze 2014** | Albumin | 24.7 | 3 |
|  | Colloid | 41.0 | 2 |
|  | Crystalloid | 84.3 | 1 |
| **Yanartas 2015**  **(Group 1)** | Albumin | 21.9 | 3 |
|  | Colloid | 48.8 | 2 |
|  | Crystalloid | 79.2 | 1 |
| **Yanartas 2015**  **(Group 2)** | Albumin | 22.2 | 3 |
|  | Colloid | 48.6 | 2 |
|  | Crystalloid | 79.2 | 1 |
| **Talvasto 2023** | Albumin | 21.5 | 3 |
|  | Colloid | 49.2 | 2 |
|  | Crystalloid | 79.3 | 1 |
| **Reduce postoperative blood loss or chest tube drainage during the first 24h** | | | |
| **Ohqvist 1981** | Albumin | 78.9 | 1 |
|  | Colloid | 33.4 | 2 |
|  | Crystalloid | 37.7 | 2 |
| **Scott 1995** | Albumin | 52.2 | 2 |
|  | Colloid | 32.5 | 2 |
|  | Crystalloid | 65.2 | 1 |
| **Tamayo 2008** | Albumin | 78.2 | 1 |
|  | Colloid | 35.3 | 2 |
|  | Crystalloid | 36.5 | 2 |
| **Cho 2014** | Albumin | 77.0 | 1 |
|  | Colloid | 34.0 | 2 |
|  | Crystalloid | 39.0 | 2 |
| **Skhirtladze 2014** | Albumin | 97.5 | 1 |
|  | Colloid | 39.0 | 2 |
|  | Crystalloid | 13.6 | 3 |
| **Yanartas 2015**  **(Group 1)** | Albumin | 77.6 | 1 |
|  | Colloid | 31.2 | 2 |
|  | Crystalloid | 41.2 | 2 |
| **Yanartas 2015**  **(Group 2)** | Albumin | 78.4 | 1 |
|  | Colloid | 33.1 | 2 |
|  | Crystalloid | 38.5 | 2 |
| **Maleki 2016** | Albumin | 66.8 | 1 |
|  | Colloid | 47.7 | 2 |
|  | Crystalloid | 35.5 | 2 |
