# Supplementary material for: Effects of albumin and crystalloid priming strategies on red blood cell transfusions in on-pump cardiac surgery: a network meta-analysis
Source: BMC Anesthesiol. 2024 Jan 16;24:26. doi: 10.1186/s12871-024-02414-y (PMC10790517; doi:10.1186/s12871-024-02414-y)
Supplement: Supplementary file 13 — Supplementary Material 13: Supplemental Table 7. Main postoperative variables of the included studies. [file 12871_2024_2414_MOESM13_ESM.docx]

**Supplemental Table 7. Main postoperative variables of the included studies.**

| **Covariate** | **Number of trials reporting the variable (%)** | **Mean Difference or Odds Ratio (95% CI)** | **I^2^ (%)** | ***P*-value** |
| --- | --- | --- | --- | --- |
| **Albumin vs Crystalloid** |  |  |  |  |
| **Postoperative Hb (g/dl)** | 2/5 (40%) | -1.05 [-1.35, -0.74] | 94 | <0.01 |
| **Ventilation time (h)** | 2/5 (40%) | 0.58 [-0.15, 1.31] | 82 | 0.12 |
| **ICU stay (d)** | 2/5 (40%) | 0.46 [-0.09,1.01] | 0 | 0.10 |
| **Hospital stay (d)** | 2/5 (40%) | -1.19 [-4.16, 1.78] | 0 | 0.43 |
| **Postoperative AKI (%)** | 4/5 (80%) | 1.37 [0.79. 2.36] | 0 | 0.26 |
| **Death (%)** | 3/5 (60%) | 1.21 [0.27, 4.00] | 13 | 0.75 |
| **Albumin vs Artificial Colloid** |  |  |  |  |
| **Postoperative Hct (%) or Hb (g/dl)** | 1/4 (33.3%) | - | - | - |
| **Ventilation time (h)** | 2/4 (50%) | 0.03 [-0.34, 0.40] | 0 | 0.88 |
| **ICU stay (d)** | 2/4 (50%) | -0.50 [-1.12, 0.12] | 0 | 0.12 |
| **Hospital stay (d)** | 2/4 (50%) | 1.13 [-1.77, 4.04] | 0 | 0.44 |
| **Postoperative AKI (%)** | 3/4 (75%) | 1.97 [0.62, 6.22] | 0 | 0.25 |
| **Death (%)** | 2/4 (50%) | 1.04 [0.18, 6.11] | 0 | 0.97 |
| **Artificial Colloid vs Crystalloid** |  |  |  |  |
| **Postoperative Hct (%) or Hb (g/dl)*** | 4/5 (80%) | -1.15 [-3.08, 0.79] | 97 | 0.24 |
| **Ventilation time (h)** | 4/5 (80%) | -0.13 [-0.51, 0.25] | 70 | 0.50 |
| **ICU stay (d)** | 3/5 (60%) | 0.42 [-0.62, 1.46] | 0 | 0.43 |
| **Hospital stay (d)** | 1/5 (20%) | - | - | - |
| **Postoperative AKI (%)** | 3/5 (60%) | 1.60 [0.52, 4.89] | 0 | 0.41 |
| **Death (%)** | 2/5 (40%) | 1.67 [0.21, 13.07] | 0 | 0.62 |

ICU, intensive care unit; AKI, acute kidney injury, Hct, Hematocrit; Hb, Hemoglobin. *If not including Hct, Hb instead, using standardized mean difference to compare characteristics.
